# Modulation of event-related desynchronization during kinematic and kinetic hand movements

**DOI:** 10.1186/1743-0003-11-90

**Published:** 2014-05-30

**Authors:** Kosei Nakayashiki, Midori Saeki, Yohei Takata, Yoshikatsu Hayashi, Toshiyuki Kondo

**Affiliations:** 1 Department of Computer and Information Sciences, Tokyo University of Agriculture and Technology, 2-24-16 Naka-cho, Koganei, 184-8588, Tokyo, Japan; 2 Hamamatsu Photonics K.K. Branch 5000, Hirakuchi, Hamamatsu, Shizuoka, Japan; 3 Brain Embodiment Laboratory, School of Systems Engineering, University of Reading, RG6 6AY, Whiteknights, Reading, UK

**Keywords:** BCI, EEG, ERD, Grasping

## Abstract

**Background:**

Event-related desynchronization/synchronization (ERD/ERS) is a relative power decrease/increase of electroencephalogram (EEG) in a specific frequency band during physical motor execution and mental motor imagery, thus it is widely used for the brain-computer interface (BCI) purpose. However what the ERD really reflects and its frequency band specific role have not been agreed and are under investigation. Understanding the underlying mechanism which causes a significant ERD would be crucial to improve the reliability of the ERD-based BCI. We systematically investigated the relationship between conditions of actual repetitive hand movements and resulting ERD.

**Methods:**

Eleven healthy young participants were asked to close/open their right hand repetitively at three different speeds (Hold, 1/3 Hz, and 1 Hz) and four distinct motor loads (0, 2, 10, and 15 kgf). In each condition, participants repeated 20 experimental trials, each of which consisted of rest (8–10 s), preparation (1 s) and task (6 s) periods. Under the Hold condition, participants were instructed to keep clenching their hand (i.e., isometric contraction) during the task period. Throughout the experiment, EEG signals were recorded from left and right motor areas for offline data analysis. We obtained time courses of EEG power spectrum to discuss the modulation of mu and beta-ERD/ERS due to the task conditions.

**Results:**

We confirmed salient mu-ERD (8–13 Hz) and slightly weak beta-ERD (14–30 Hz) on both hemispheres during repetitive hand grasping movements. According to a 3 × 4 ANOVA (speed × motor load), both mu and beta-ERD during the task period were significantly weakened under the Hold condition, whereas no significant difference in the kinetics levels and interaction effect was observed.

**Conclusions:**

This study investigates the effect of changes in kinematics and kinetics on resulting ERD during repetitive hand grasping movements. The experimental results suggest that the strength of ERD may reflect the time differentiation of hand postures in motor planning process or the variation of proprioception resulting from hand movements, rather than the motor command generated in the down stream, which recruits a group of motor neurons.

## Background

In recent years many countries are faced with aged society. As growth of the elderly population continues, the number of stroke patients with motor paralysis increases [1]. Brain-Computer Interfaces (BCIs) have been suggested as one of effective neurorehabilitation means for the stroke patients, because it can be closing the impaired sensorimotor loop by compensating somatosensory feedback on their motor attempt [2-4]. The experience of the BCI neurorehabilitation should be important for promoting brain neuroplasticity to recover from motor paralysis.

A significant factor for a successful BCI neurorehabilitation is reliable detection of human motor intent. Neurophysiological studies have demonstrated that not only neuronal spike recordings but also local field potential (LPF) in cortical [5] and sub-cortical [6,7] areas can be used to decode the movements. In particular, event-related power changes in the neural oscillatory activities have been studied in relation of sensorimotor processes. For example, LFP in the subthalamic nucleus (STN) during voluntary grip with different motor efforts has been investigated [7]; they reported that power suppression in the beta band (13–30 Hz) and power increase in the theta/alpha (4–12 Hz), the gamma (55–90 Hz) and higher frequency bands were observed during motor execution, and the power changes correlated with effort levels. Engel et al. (2010) reported that the beta band activity is attenuated by voluntary movements, but is increased during steady contractions; thus they suggested that the beta band oscillations may reflect maintenance of status quo in both sensory and motor circuits [6].

The event-related and frequency-band specific power decrease/increase are known as event-related desynchronization (ERD) and synchronization (ERS). For the practical use of BCI, attempts to decode the oscillatory activities associated with human sensorimotor processes in non-invasive manner have been intensively investigated [8-13]. Those research are mainly focused on the ERD/ERS in alpha and beta bands. The salient brain oscillations in alpha band (8–13 Hz) over the sensorimotor area is known as mu-rhythm, which is desynchronized during motor planning, execution and imagery of hand/finger movements [8,12,13], even when one observes the movement by others [11], though it does not coincide with the results of LPF in STN [7]. Whereas the sensorimotor beta power (14–30 Hz) is totally consistent with invasive studies in the above [6,7], it is attenuated by a voluntary execution and imagery of hand/foot movements, and even passive movements [14], but it prominently increases after movement offset (known as beta rebound) and during steady contractions [13,15].

Because the ERD can be observed in both alpha and beta bands during voluntary motor execution and imagery, it is expected to be an intuitive and self-paced (i.e., asynchronous) BCI [16,17]. However it is suggested that the ability of voluntary ERD generation varies with individual and it is difficult for most novice BCI users [10]; thus it is generally agreed that sufficient neurofeedback training is necessary to utilize this type of BCI [18,19].

Understanding the conditions (and underlying brain mechanism) which causes a significant ERD would be crucial to improve the reliability of the ERD-based BCI. In this context, Cassim et al. (2000) investigated the relationship between movement durations under brief/sustained wrist extensions and resulting ERD; they reported there was no difference in pre and post movement periods [20]. Jeon et al. (2011) reported consistent movement duration effect in their motor imagery study [21]. Yuan et al. (2010) reported that mu and beta-ERD are correlated with the speed of repetitive hand grasping movements in both actual execution and motor imagery conditions [12]. Stancak et al. (1997) reported that post movement mu-ERD and beta-ERS under the heaviest external load condition showed longer duration, concluding that the ERD/ERS is influenced by external load opposing finger movements [22]. In contrast, Chakarov et al. (2009) reported that EEG and EMG spectral power did not show any significant difference among the three force conditions, but the beta range EEG-EMG coherence increases as the load increases [15].

Even though several works investigated the effect of kinematics and kinetics on resulting ERD, the sensorimotor processes the ERD really reflects and its frequency band specific role have not been agreed and are under investigation. In the paper, we systematically investigated the effects of kinematics (speeds) and kinetics (motor loads) during repetitive hand grasping movements on resulting mu and beta-ERD to be clear some controversial points in the previous literature. Accordingly we discuss about brain function in terms of human sensorimotor execution process.

## Methods

### Participants

Eleven healthy young participants aged 19–23 years (mean age, 21.1 years) took part in the following experiment. All were right handed and had no record of any neurological disorders. The recruitment of the participants and the experimental procedure were approved by the ethics committee of the Tokyo University of Agriculture and Technology. All the participants were informed the aim and the procedure of experiments and provided written informed consent prior to participate a series of trials under supervision.

### Experimental environment

During the experiment, the participants wore an EEG cap with electrodes; they were seated in a comfortable high-back chair and placed their right arm on the armrest so that a group of muscles of their upper limb is relaxed against the gravity. An LCD monitor was located in front of them and they could see a visual cue on the display.

### Experimental design

**Figure 1:**
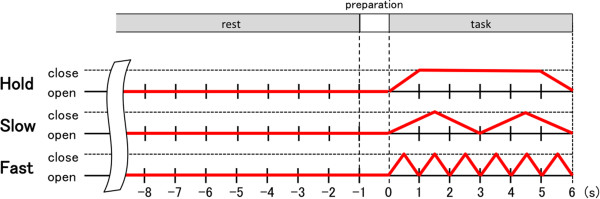
**Kinematics pattern of the instructed hand grasping movement.** Participants were instructed to relax during the rest and preparation periods, whereas they were asked to grasp their right hand at three distinct speeds (i.e., Hold, Slow, and Fast) and four different grasping loads (0, 2, 10, and 15 kgf) during the task period.

We executed an experiment to investigate how the kinematic and kinetic changes in hand grasping movements affect the resulting ERD strength. Participants were asked to close/open their right hand repetitively at three different speeds (Hold, Slow (1/3 Hz), and Fast (1 Hz)) and four distinct grasping loads (0, 2, 10, and 15 kgf). To constrain the maximum grasping force during the task, we used three different hand grips which have the different values of grasping load to the subjects without particular visual information indicating the difference of load. In order to avoid the effect of muscle fatigue, the twelve experimental conditions (i.e., 3 speeds and 4 grasping loads) were conducted in a fixed order for all participants, namely starting from no load and three different speed conditions (Hold, Slow, and Fast in this order), then the grasping load condition was changed to be one rank heavier.In each experimental condition, the participants repeated 20 experimental trials, each of which consisted of rest, preparation, and task periods, respectively. To avoid anticipatory response, a random time duration from 8 to 10 s was set in the rest period. Immediately after the rest period, a visual cue (a colored filled circle) was displayed on the LCD monitor to notify the participants of the preparation period (1 s). Participants were instructed to move their hand paced by the visual cue which was periodically moving up and down in vertical direction indicating closing/opening hand during the task period of 6 s. Figure 1 represents the instructed hand kinematics during an experimental trail. Note that participants had to keep clenching their hand (4 s) under the Hold condition.

### EEG recording

**Figure 2:**
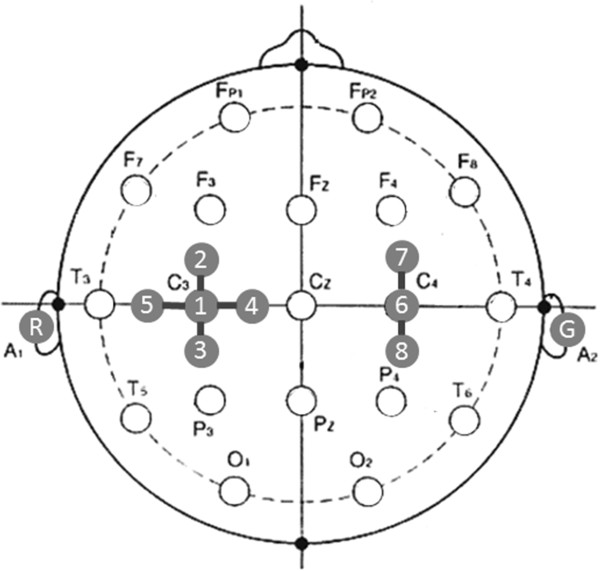
**Layout of EEG electrodes.** Eight active dry EEG electrodes were placed C3/C4 and the surrounding area based on the international 10–20 system. In offline analysis EEG data were re-treated by bipolar spatial derivation between C3/C4 and the neighbor electrodes.

To focus on the oscillatory activities in primary motor area, we recorded EEG signals from eight active dry electrodes (g.SAHARA electrode, g.tec, Vienna, Austria) placed around the C3 (left hemisphere) and C4 (right hemisphere) of the international 10–20 system. These areas are well known that 1) mainly reflecting contralateral hand movement/imagery and 2) activated bilaterally in the case of actual hand movements [12]. The active dry electrodes is a latest technology, and its availability is validated in [23] and [24]. The configuration of eight electrodes are shown in Figure 2. Five electrodes were located in the cross-configuration with C3 at the center, whereas three electrodes were located in line C4 at the center and other two in anterior and posterior location. The distance between them was kept at 35-mm in each configuration. The reference and ground electrodes were placed at A1 and A2 (i.e., left and right mastoids), respectively.

The EEG signal was sampled at 512 Hz, preamplified in a specific electrode box (g.SAHARAbox, g.tec) and amplified using a digital multi-telemeter system (WEB5000, NIHON KOHDEN, Tokyo, Japan). The EEG data were band-passed from 0.3 to 100 Hz in the amplifier. The analog signal was converted into digital data by an AD converter board (LPC-321416, Interface, Japan), and stored in a personal computer (Windows 7, Core i5-760, 2.8 GHz).

### Signal processing

In offline analysis EEG data were re-treated by bipolar spatial derivation between C3 (channel 1) and other nearest neighbor electrodes (channels 2–5), and were identified as Ch1–2, Ch1–3, Ch1–4, and Ch1–5; in the same manner, the signals by bipolar derivation with respect to C4 (channel 6) were identified as Ch6–7 and Ch6–8 (see Figure 2).

To calculate ERD as a function of time, the time window of 1 s (i.e., 512 samples) was employed to perform the short time Fourier transform (STFT), and the time window was shifted by 1/16 s, we thus obtained instantaneous power spectrum (*P*_*n*_) at every time window. The ERD was defined as a percentage of power decrease in a specific frequency band relative to the baseline period (i.e., the rest period). We calculated the relative power (RP) using the instantaneous power spectrum (*P*_*n*_):

$$\begin{array}{l} P_{\text{rest}}=\frac{1}{\left|T_{\text{rest}}\right|}\underset{n\in T_{\text{rest}}}{\sum }P_{n},\\ P_{\text{task}}=\frac{1}{\left|T_{\text{task}}\right|}\underset{n\in T_{\text{task}}}{\sum }P_{n},\\ \text{RP}(n)=\frac{P_{n}-P_{\text{rest}}}{P_{\text{rest}}}\times 100\\ \text{RP}=\frac{P_{\text{task}}-P_{\text{rest}}}{P_{\text{rest}}}\times 100 \end{array}$$

where *P*_*rest *_and *P*_*task *_are the mean power spectra during the rest period (*T*_*rest*_) and the task period (*T*_*task*_), respectively. *RP* was averaged across trials within a participant. To obtain salient mu and beta-ERD individually, we evaluated the most significant frequency bin (3 Hz band width) and derivation channel (for both C3 and C4) at every experimental condition. The frequency range searching for the salient mu and beta-ERD were selected from 8 to 13 Hz and from 14 to 30 Hz, respectively.

## Results

### Time course of ERD/ERS

**Figure 3:**
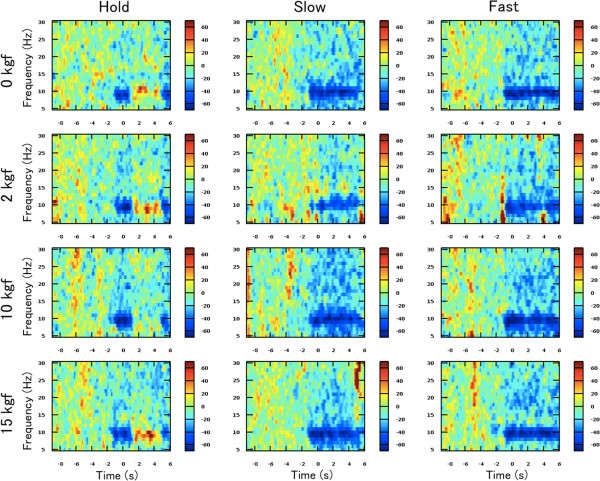
**Time course of ERD/ERS.** Each figure shows a time course of the relative power decrease (ERD) and increase (ERS) on C3 under each speed and motor load condition. This is a typical result of a participant (Subject G). The horizontal axis indicates the time aligned at the onset of the task period (0 s), and the vertical axis indicates the frequency. The colorbar indicates the percentage of ERD/ERS. The mu-ERD can be observed during the task period. In the Hold condition, the mu-ERD disappears and mu-ERS are alternatively confirmed in the middle of the task period.

Figure 3 illustrates the time courses of the percentage change in relative power (i.e. *RP *(*n*)) on C3 of a typical participant (subject G) under each speed and grasping load condition. In the figure, significant mu-ERD (i.e., 8–13 Hz) and slightly weak beta-ERD (14–30 Hz) right after the visual cue onset (-1 s in the horizontal time scale) can be observed.

Moreover in the Hold condition, the mu and beta-ERD was changed into mu-ERS immediately after hand closing, regardless of the motor load (i.e., isometric contraction) conditions. This tendency was commonly observed in all the other participants.

### Effect of kinematics and kinetics of hand movement on resulting ERD

**Figure 4:**
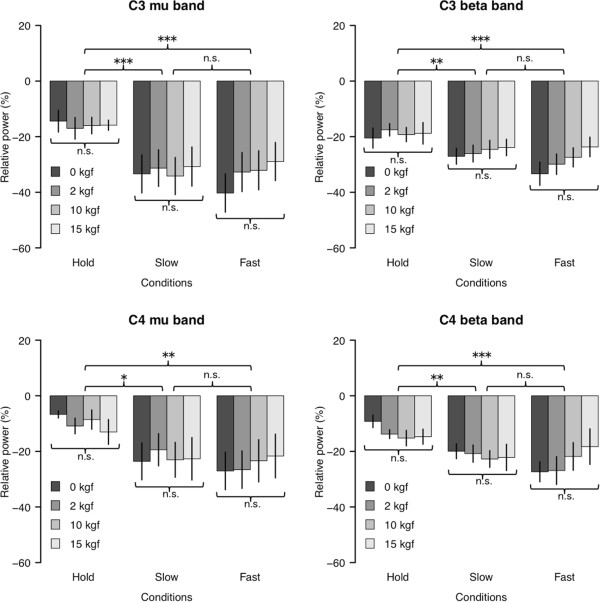
**Effects of kinematics and kinetics on the resulting mu and beta-ERD over the contralateral (C3) and ipsilateral (C4) motor areas.** Each figure demonstrates the statistical comparisons of the relative power decrease (ERD) during the task period under the different speeds and loads conditions averaged across subjects (n=11). Statistically significant difference was confirmed between the Hold and other speed conditions. On the other hand, there were no significant difference among kinetics conditions. ***(*p *< 0.001), **(*p *< 0.01), *(*p *< 0.05), and n.s. (*p *> 0.1).

Figure 4 demonstrates the mean and the standard error of the relative power (*RP*) in C3/C4 and mu/beta frequency bands across all the participants (n=11) under the different grasping speeds and loads condition. Participant-specific bipolar channels (C3/C4) and frequency ranges (mu/beta bands) used for the statistical evaluation are listed in Table 1.

**Table 1:** Participants-specific bipolar channels (C3/C4) and frequency bands used for the statistical evaluation of mu and beta-ERD

**mu**										
			**0 kgf**		**2 kgf**		**10 kgf**		**15 kgf**	
**C3**	**Age**	**Sex**	**Ch**	**Freq.**	**Ch**	**Freq.**	**Ch**	**Freq.**	**Ch**	**Freq.**
A	19	M	1-2	11–13	1-2	11–13	1-2	11–13	1-2	11–13
B	20	M	1-3	8–10	1-3	9–11	1-3	10–12	1-5	8–10
C	22	F	1-2	11–13	1-4	11–13	1-4	11–13	1-3	8–10
D	23	M	1-3	10–12	1-3	9–11	1-3	11–13	1-4	9–11
E	20	F	1-3	11–13	1-3	11–13	1-2	11–13	1-3	11–13
F	21	M	1-3	9–11	1-3	8–10	1-3	11–13	1-2	8–10
G	22	M	1-3	10–12	1-3	11–13	1-3	10–12	1-3	10–12
H	19	M	1-2	11–13	1-2	11–13	1-3	11–13	1-3	11–13
I	23	M	1-2	11–13	1-2	11–13	1-3	11–13	1-3	11–13
J	21	M	1-2	11–13	1-3	11–13	1-3	11–13	1-5	11–13
K	22	M	1-4	11–13	1-4	8–10	1-3	11–13	1-3	8–10
**C4**	**Age**	**Sex**	**Ch**	**Freq.**	**Ch**	**Freq.**	**Ch**	**Freq.**	**Ch**	**Freq.**
A	19	M	6-8	9–11	6-7	11–13	6-7	8–10	6-7	11–13
B	20	M	6-8	9–11	6-7	8–10	6-7	8–10	6-8	9–11
C	22	F	6-8	11–13	6-7	11–13	6-8	11–13	6-8	8–10
D	23	M	6-8	11–13	6-7	11–13	6-8	11–13	6-7	10–12
E	20	F	6-8	11–13	6-7	11–13	6-7	11–13	6-7	11–13
F	21	M	6-8	9–11	6-7	9–11	6-8	10–12	6-7	11–13
G	22	M	6-8	10–12	6-8	10–12	6-8	10–12	6-8	10–12
H	19	M	6-8	9–11	6-7	11–13	6-7	11–13	6-8	11–13
I	23	M	6-7	11–13	6-7	10–12	6-8	11–13	6-8	11–13
J	21	M	6-8	10–12	6-7	11–13	6-8	11–13	6-8	11–13
K	22	M	6-8	8–10	6-8	10–12	6-7	11–13	6-7	9–11
**beta**										
			**0 kgf**		**2 kgf**		**10 kgf**		**15 kgf**	
**C3**	**Age**	**Sex**	**Ch**	**Freq.**	**Ch**	**Freq.**	**Ch**	**Freq.**	**Ch**	**Freq.**
A	19	M	1-2	14–16	1-2	16–18	1-2	16–18	1-3	23–25
B	20	M	1-2	23–25	1-4	16–18	1-3	16–18	1-3	15–17
C	22	F	1-2	14–16	1-2	14–16	1-4	14–16	1-3	14–16
D	23	M	1-3	20–22	1-2	24–26	1-3	24–26	1-2	25–27
E	20	F	1-2	14–16	1-2	14–16	1-2	14–16	1-3	14–16
F	21	M	1-3	14–16	1-2	15–17	1-4	20–22	1-2	22–24
G	22	M	1-3	14–16	1-5	19–21	1-4	14–16	1-3	14–16
H	19	M	1-2	14–16	1-2	16–18	1-2	14–16	1-2	18–20
I	23	M	1-2	21–23	1-3	23–25	1-3	22–24	1-3	20–22
J	21	M	1-4	14–16	1-4	17–19	1-2	23–15	1-5	14–16
K	22	M	1-4	26–28	1-4	26–28	1-5	15–17	1-2	27–29
**C4**	**Age**	**Sex**	**Ch**	**Freq.**	**Ch**	**Freq.**	**Ch**	**Freq.**	**Ch**	**Freq.**
A	19	M	6-8	14–16	6-7	14–16	6-7	14–16	6-7	14–16
B	20	M	6-8	16–18	6-8	16–18	6-8	19–21	6-7	14–16
C	22	F	6-8	14–16	6-8	14–16	6-8	14–16	6-7	14–16
D	23	M	6-8	17–19	6-7	28–30	6-8	21–23	6-7	23–25
E	20	F	6-8	14–16	6-7	14–16	6-7	14–16	6-7	14–16
F	21	M	6-8	27–29	6-8	22–24	6-7	21–23	6-7	14–16
G	22	M	6-7	24–26	6-7	19–21	6-7	24–26	6-7	14–16
H	19	M	6-8	28–30	6-7	14–16	6-8	14–16	6-8	17–19
I	23	M	6-7	20–22	6-7	21–23	6-7	23–25	6-7	15–17
J	21	M	6-7	18–20	6-8	19–21	6-7	14–16	6-7	15–17
K	22	M	6-8	26–28	6-7	17–19	6-7	20–22	6-7	28–30

Channel 1 represents the C3, and channels 2, 3, 4 and 5 indicate the anterior, posterior, right and left of C3. Channel 6 represents the C4, and channels 7 and 8 indicate the anterior and posterior of C4.

A 3 × 4 repeated-measures ANOVA (speed × motor load) was applied to each hemisphere (C3/C4) and frequency band (mu/beta). Under the case of C3 and mu-rhythm, a significant main effect in grasping speed (*F *(2,120) = 11.214, *p *< 0.001) whereas neither the effect of motor load (*F *(3,120) = 0.250, *p *= 0.862) nor interaction effect (*F *(6,120) = 0.259, *p *= 0.955) was confirmed. More importantly, Tukey’s HSD for multiple comparisons exhibits a significant difference between Hold and the other speed conditions (Hold–Slow: *p *< 0.001, Hold–Fast: *p *< 0.001). In all the cases, tendency of the statistical analyses were identical as shown in the figure.

## Discussion

In the present study, we investigated the modulation of event-related and frequency-band specific power decrease/increase (i.e., mu and beta ERD/ERS) elicited by actual hand grasping movements under various kinematics (three different velocity patterns) and kinetics (four distinct motor loads) conditions. Our results demonstrated that (1) both time-averaged mu and beta-ERD levels during actual movement period were significantly weakened under the Hold condition and (2) there was no significant difference among different kinetics conditions. Note that the hand grasping movements under the Hold condition included isometric contraction in the middle phase of the task period (i.e., 1–5 s) whereas the movements in the other phases and speed conditions were isotonic contraction. This implies that the time differentiation in kinematics (change of hand posture) is correlated with the strength of mu/beta-ERD but maintenance of the current sensorimotor state (i.e., keeping the hand posture in the Hold condition) was related to the mu/beta rebound. These results seem to be consistent with (Engel and Fries, 2010) [6]. Moreover our results suggested that the modulation of the ERD/ERS may not depend on the muscle activities to resist the motor loads.

The relationship between the modulation of ERD/ERS and the motor efforts has been investigated in several relevant studies. Kilavik et al. (2013) stated that during stable object holding, beta oscillations display a relative increase in power and are phase synchronized with the EMG of the tonically contracting muscles [13]. Stancak et al. (1997) used a finger lifting movement against several motor loads as the motor task, and reported that the duration of mu-ERD (not the time-averaged mu-ERD level) was significantly longer under the most heavy load condition and that post-movement beta-synchronization was also longer under the heaviest load as compared to the no-load condition, concluding that the ERD/ERS is influenced by external load [22]. Tan et al. (2003) reported that neural activity in sub-cortical area was linked to the motor efforts, in their neurophysiological research [7]. Furthermore functional MRI studies indicated that cortical BOLD signal may correlate with the grasping force levels [25,26]. On the other hand, Pistohl et al. (2013) suggested that grasping force did not affect the amplitude of movement-related power decrease in their ECoG study [5]. In the study they employed the participants undergoing pre-neurosurgical diagnosis, and the invasive method, ECoG from the human motor cortex could successfully distinguish two different grasp types (precision vs. whole-hand grip) even if the weights of the manipulating objects were different. The task used in our study is also hand grasping; thus we suggest that the ERD might be insensitive to the change in kinetics. To our knowledge as the closest piece of work to our results and indication, Chakarov et al. (2009) reported that EEG and EMG spectral power did not show any significant difference among the three force conditions, though the beta range EEG-EMG coherence increases as the load increases [15].

Even though the above works appears to be controversial, the modulation of ERD/ERS may be dependent on several factors such as 1) experimental paradigm, for example single execution or repetitive motion 2) frequency range 3) how the average is performed over task duration and 4) brain regions; invasive or non-invasive. Our experimental protocol allows us to systematically discuss the effects of kinematics and kinetics on resulting ERD, and clarify some controversial points in previous literature in the systematic experimental conditions in terms of 1) isometric and isotonic contraction condition, 2) time averaging of ERD/ERS over task period, and 3) kinematics patterns (speed) of grasping motion.

Human sensorimotor process consists of sub-processes; motor intention, planning of motion trajectory, motor command generation, and receiving sensory feedback. Our findings showed that the ERD might not reflect the strength of motor load. However motor commands should be continuously generated to maintain the hand grasping (isometric contraction). Thus we consider that the motor command generation process have little effect on the resulting mu and beta ERD. In contrast, changes in hand posture were found out to be correlated with the ERD strength. We suggest that the strength of mu and beta-ERD may reflect the time differentiation of hand postures in our motor planning process or the variation in proprioceptive sensation resulting from the hand movements rather than the motor command generated in the down stream, which recruits a group of motor neurons.

## Abbreviations

BCI: Brain-computer interface; EEG: Electroencephalogram; ERD: Event-related desynchronization.

## Competing interests

The authors declare that they have no competing interests.

## Authors’ contributions

KN and TK supervised the study, designed the experiment, developed the experimental system, signal processing and statistical analysis. TK and YH contributed to discussion of the obtained results. KN, TK, and YH wrote the manuscript. MS, YT, and YH provided fruitful comments for the experimental procedure and analysis. All the authors have read and approved the final manuscript.
